# Activation of voltage-gated KCNQ/Kv7 channels by anticonvulsant retigabine attenuates mechanical allodynia of inflammatory temporomandibular joint in rats

**DOI:** 10.1186/1744-8069-6-49

**Published:** 2010-08-27

**Authors:** Wen Xu, Yuwei Wu, Yeping Bi, Lei Tan, Yehua Gan, KeWei Wang

**Affiliations:** 1 Department of Neurobiology, Neuroscience Research Institute, Peking University Health Science Center, 38 Xueyuan Road, Beijing 100191, China; 2 Center for TMD & Orofacial Pain, Peking University School and Hospital of Stomatology, 22 Zhongguancun Nandajie, Haidian District, Beijing 100081, China

## Abstract

**Background:**

Temporomandibular disorders (TMDs) are characterized by persistent orofacial pain and have diverse etiologic factors that are not well understood. It is thought that central sensitization leads to neuronal hyperexcitability and contributes to hyperalgesia and spontaneous pain. Nonsteroidal anti-inflammatory drugs (NSAIDs) are currently the first choice of drug to relieve TMD pain. NSAIDS were shown to exhibit anticonvulsant properties and suppress cortical neuron activities by enhancing neuronal voltage-gated potassium KCNQ/Kv7 channels (M-current), suggesting that specific activation of M-current might be beneficial for TMD pain.

**Results:**

In this study, we selected a new anticonvulsant drug retigabine that specifically activates M-current, and investigated the effect of retigabine on inflammation of the temporomandibular joint (TMJ) induced by complete Freund's adjuvant (CFA) in rats. The results show that the head withdrawal threshold for escape from mechanical stimulation applied to facial skin over the TMJ in inflamed rats was significantly lower than that in control rats. Administration of centrally acting M-channel opener retigabine (2.5 and 7.5 mg/kg) can dose-dependently raise the head withdrawal threshold of mechanical allodynia, and this analgesic effect can be reversed by the specific KCNQ channel blocker XE991 (3 mg/kg). Food intake is known to be negatively associated with TMJ inflammation. Food intake was increased significantly by the administration of retigabine (2.5 and 7.5 mg/kg), and this effect was reversed by XE991 (3 mg/kg). Furthermore, intracerebralventricular injection of retigabine further confirmed the analgesic effect of central retigabine on inflammatory TMJ.

**Conclusions:**

Our findings indicate that central sensitization is involved in inflammatory TMJ pain and pharmacological intervention for controlling central hyperexcitability by activation of neuronal KCNQ/M-channels may have therapeutic potential for TMDs.

## Background

Temporomandibular disorders (TMDs) are an assortment of clinical conditions characterized by pain in the temporomandibular joint (TMJ) and/or the masticatory muscles [1]. The main symptoms exhibited by TMD patients include orofacial pain, altered jaw mechanics, impaired masticatory function and sounds from the TMJ, with few or no peripheral tissue abnormalities [2]. As TMD pain persists over time, it is thought that changes in the central nervous system (CNS) lead to altered neuronal processing in the brain, with central sensitization and hyperexcitability, ultimately affecting perception of TMD pain [3,4]. Accumulating clinical evidence shows that patients with TMDs have generalized hypersensitivity of CNS nociceptive pathways, resulting in amplification of minimal nociceptive stimuli arising from the peripheral tissues [1,5].

TMDs are often managed clinically by modifying drug regimens to achieve desired therapeutic end points, but treatment of TMDs remains a clinical challenge because its diverse etiologic factors are not well understood [1,3,4]. Although anticonvulsants which act on molecular target(s) in the brain are primarily intended to prevent epileptic seizures by suppressing neuronal hyperexcitability [1,6,7], they are often prescribed "off-label" for treatment of TMDs, suggesting central intervention to suppress hyperexcitability may be an effective clinical approach. The anticonvulsant gabapentin that binds to α2δ, an auxiliary subunit of voltage-gated calcium channels, can significantly relieve TMD pain compared to placebo groups [8], providing clinical evidence that central hyperexcitability is involved in the generation of TMD pain [5].

The anticonvulsant retigabine which was discovered in the 1980 s has been shown to attenuate inflammatory and neuropathic pain in rodent animal models [9-13], and to decrease neuronal excitability of noceiceptive neurons and C-type nerve fibers [14,15]. Retigabine is best described for its novel mechanism of action that involves specific activation of neuronal M-current encoded by Kv7.2-7.5 voltage-gated potassium channels expressed in various neurons of the peripheral and central nervous systems [16-19]. The M-current is a subthreshold voltage-gated K^+ ^current that serves as a brake to suppress abnormal ectopic discharges of neurons and control neuronal hyperexcitability [14,15,20-22].

Nonsteroidal anti-inflammatory drugs (NSAIDs) that act as nonselective inhibitors of COXs (cyclooxygenases) are widely used as first-line drugs for TMD treatment [7,23], but little is understood about their mechanism of action for the relief of TMD pain. Two NSAIDs, diclofenac sodium and meclofenamic acid, were shown to exhibit anticonvulsant activities by activation of the KCNQ/Kv7 channel [24], suggesting that opening KCNQ/Kv7 channels may be beneficial for pain relief of TMDs. Therefore, we hypothesized that central suppression of neuronal hyperexcitability by activation of central KCNQ/Kv7 channel function may lead to reverse the pain of inflamed TMJs. To evaluate this concept, we took advantage of the centrally acting retigabine and investigated its effect on mechanical allodynia in inflammed temporomandibular joints induced by complete Freund's adjuvant (CFA) in rats. In this study, we found that retigabine can dose-dependently elevate the pain threshold of TMJ inflammation induced by CFA, and this effect was antagonized by the specific channel blocker XE991. The characteristic reduction of food intake secondary to TMJ dysfunction induced by the inflammation also was reversed by retigabine in a dose-dependent manner. Our findings for the first time indicate that the anticonvulsant compound retigabine which activates KCNQ/Kv7 channels may provide a potential treatment for TMD.

## Results

### Inflamed TMJ induced by CFA

**Figure 1:**
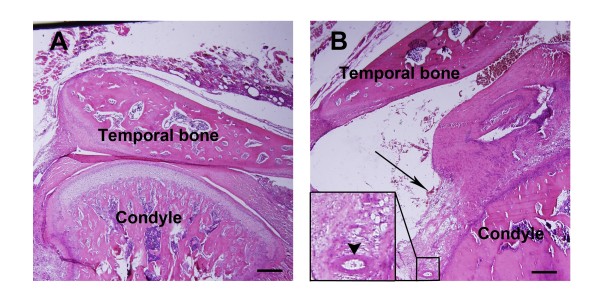
**Histopathologic examination of inflammatory TMJ region induced by CFA in rats**. A. Photomicrograph of TMJ 24 hours after injection of saline as a normal control. B. Photomicrograph of TMJ 24 hours after injection of CFA. Exuberant hypertrophy of the synovial tissue, angiogenesis in the enlarged area (indicated by arrowhead) and fibrin-like exudates (indicated by arrow) in the joint space were observed (Magnification × 4, scale bar = 200 μm).

We started by validating CFA induces TMJ inflammation in rats. As previously described, the injection of CFA into the temporomandibular joint (TMJ) induced significant mechanical hypersensitivity in the TMJ region associated with inflammation [25-27]. Twenty-four hours after the CFA injection in the TMJ, chromodacryorrhea in the eyes and intense redness and swelling over the TMJ region were observed in all CFA-injected groups, but were not found in the control group. The injection site was anatomically confirmed by dissection of the TMJ region. Histopathologic examination showed the synovial tissues were hypertrophied with an increase of infiltrating leucocytes in the TMJ following CFA injection [28,29], while there were no such changes in the control TMJ without CFA (Figure 1). Angiogenesis and fibrin-like exudate in the superior joint space were also observed in the CFA injected TMJ as compared to the control joint (Figure 1), indicating that injection of CFA into the TMJ successfully induced inflammation.

**Figure 2:**
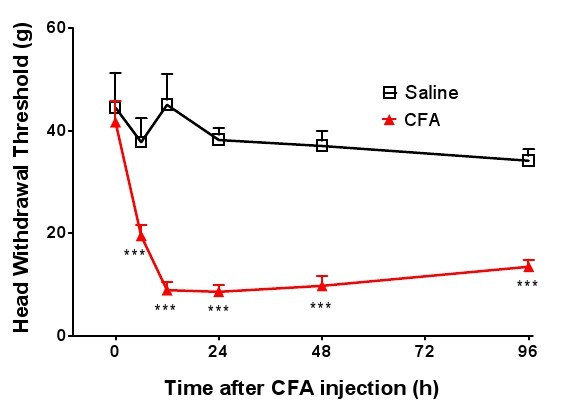
**Development of mechanical allodynia in TMJ induced by CFA in rats**. Head withdrawal threshold was measured using an electric von Frey anesthesiometer over a 96-hour period for the saline control group (open squares) and the CFA group (filled triangles). CFA was injected at time 0 and the withdrawal threshold was measured at time points 6 h, 12 h, 24 h, 48 h, and 96 h. The lowest head withdrawal threshold occurred 12 h after CFA injection and was maintained for about 36 h (*P *< 0.001 for time period of 12 to 96 hours, compared with the saline group, two-way ANOVA).

In behavioral tests, the mechanical head withdrawal threshold was measured at time points 6, 12, 24, 48 and 96 hours after CFA injection into the TMJ region. Mechanical hypersensitivity developed and reached the lowest head withdrawal threshold at 12 hours (Figure 2), and mechanical hypersensitivity was maintained for at least 4 days, compared with saline group (Figure 2). This result confirmed the relationship of inflammation in the TMJ induced by CFA and mechanical hypersensitivity, and this model was used for the entire study.

### Attenuation of mechanical allodynia of inflamed TMJ by activation of KCNQ/Kv7 with retigabine

**Figure 3:**
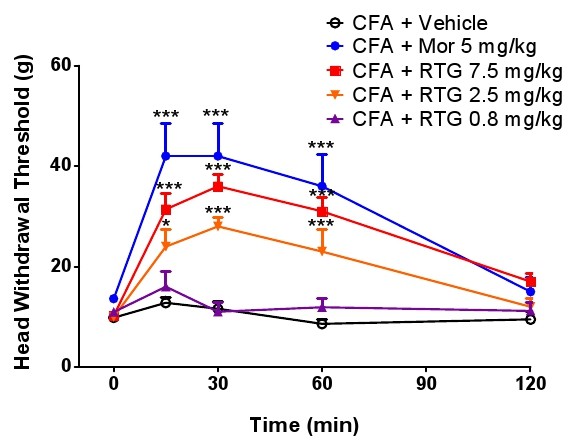
**Attenuation of mechanical TMJ allodynia by anticonvulsant retigabine in a dose-dependent manner**. Drugs (Mor: morphine and RTG: retigabine) of different concentrations were administered to rats with TMJ inflammation by intraperitoneal (i.p) injection at time 0. The withdrawal threshold was measured at 15, 30, 60 and 120 min after drug treatment, with morphine as a positive control. Statistical significance of *p *< 0.05 (two-way ANOVA followed by Bonferroni *post hoc*) was obtained for the period 15-60 min for the groups CFA + morphine (5 mg/kg), and CFA + retigabine (7.5 mg/kg or 2.5 mg/kg), as compared with the group CFA + vehicle.

To test whether retigabine administration can attenuate the mechanical allodynia of inflamed TMJ, we first measured the analgesic effect of morphine on the inflamed TMJ pain. Morphine (5 mg/kg, i.p.) as a positive control reversed the decreased head withdrawal threshold of rats with inflamed TMJ (Figure 3). We then tested the effect of retigabine on the head withdrawal threshold. Intraperitoneal administration of retigabine resulted in dose-dependent attenuation of mechanical TMJ allodynia (Figure 3). The effect of retigabine on inhibition of mechanical hypersensitivity was observed 15 min after retigabine injection, and lasted for about 90 min (Figure 3).

**Figure 4:**
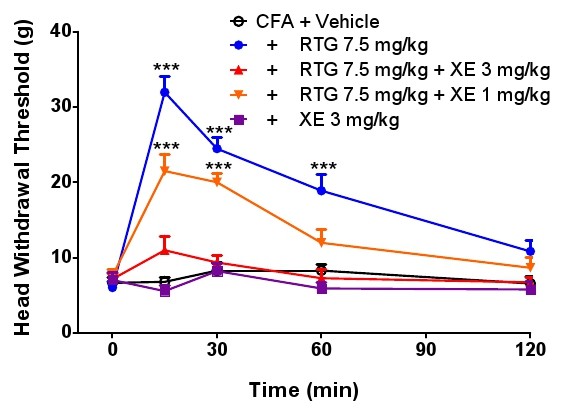
**Retigabine reduced allodynia reversed by the blocker XE991**. RTG (retigabine) at 7.5mg/kg attenuated the inflamed TMJ allodynia, and the effect was reversed by XE991 (3 mg/kg or 1 mg/kg) in a dose- and time-dependent manner. XE991 alone (3 mg/kg) had no effect on the head withdrawal threshold in TMJ inflammation. Statistical significance of *p *< 0.05 (two-way ANOVA followed by Bonferroni post-test) was obtained for the period 15-60 min for the group CFA + RTG 7.5 mg/kg, as compared with CFA + vehicle; and was also obtained for the two groups CFA + RTG 7.5 mg/kg + XE 3 mg/kg, and CFA + RTG 7.5 mg/kg + XE 1 mg/kg, as compared with the group of CFA + RTG 7.5 mg/kg. XE: XE991, RTG: retigabine. The plus sign (+) in the legend indicates CFA treatment.

**Figure 5:**
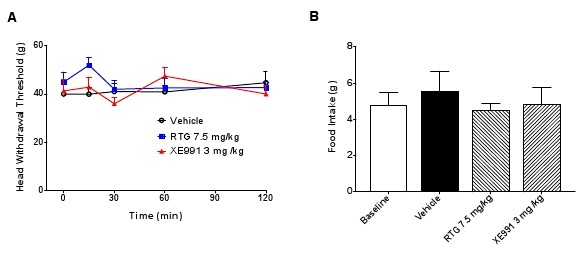
**Head withdrawal threshold and food intake in normal rats was not affected by retigabine and XE991**. (A), RTG (retigabine) at 7.5 mg/kg and XE991 at 3 mg/kg had no effect on head withdrawal threshold of normal rats without TMJ inflammation, as compared with the vehicle (two-way ANOVA followed by Bonferroni post-test). (B), Food intake values in normal rats without TMJ inflammation. During the 2 h period of food intake, retigabine (RTG) and XE991 had no effect on normal food intake in rats without TMJ inflammation. The food intake value in the baseline group (4.8 g) indicates the normal value obtained before drug or vehicle administration. The food intake values in the groups treated by retigabine (7.5 mg/kg, i.p.) and XE991 (3 mg/kg, i.p.) were 4.5 g and 4.8 g, respectively. The food intake value of the vehicle group was 5.5 g. There was no statistical significance between these four groups (one-way ANOVA followed by Dunnett's test).

It is known that the specific KCNQ/Kv7 channel blocker XE991 can reverse the effect of retigabine on inflammatory and neuropathic pain [9-11]. To further confirm the specific action of retigabine on TMJ inflammatory pain through activation of KCNQ/Kv7 channel function, we used the blocker XE991 to test whether the effect of retigabine could be reversed. Figure 4 shows that the effect of retigabine (7.5 mg/kg) on head withdrawal threshold can be antagonized by XE991 (either 3 mg/kg or 1 mg/kg), compared with the vehicle control in the presence or absence of XE991 (3 mg/kg), indicating that retigabine reduction of allodynia in the inflamed TMJ can be reversed by this blocker. As a control, we also tested both retigabine and XE991 for effect on head withdrawal threshold in normal rats. The result showed that neither retigabine nor XE991 have any effect on the baseline head withdrawal threshold, compared with the vehicle group (Figure 5A).

### Food intake with inflamed TMJs was increased by activation of KCNQ/Kv7 with retigabine

To further confirm the effect of retigabine, we also measured food intake. The amount of food intake is negatively associated with TMJ pain due to the limited movement of the TMJ after inflammation [30,31]. After injection of CFA into TMJ, rats were kept one per cage and supplied with water for 12 h, but were not given food. The rat was then given food but no water, and the amount of food consumed during a 2 h period was recorded as the food intake. Without induction of TMJ inflammation, normal rats consumed about 4 grams of food, and the same amount of food was consumed by the vehicle, retigabine (7.5 mg/kg) and XE991 groups (3 mg/kg), indicating that neither retigabine nor XE991 has any effect on basal consumption of food in normal rats (Figure 5B).

**Figure 6:**
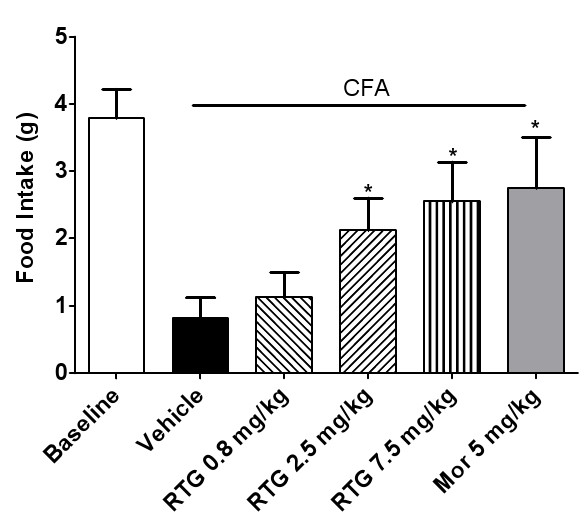
**Increased food intake with retigabine administration in rats with TMJ inflammation injected with CFA**. The food intake in the baseline group was measured in normal rats without CFA injection. Twenty-four hours after CFA injection, the effect of retigabine or morphine as a positive control on food intake was measured, and compared with the vehicle (CFA alone) group. Retigabine enhanced the food intake in dose-dependent manner, as compared with the vehicle. Statistical significance was obtained between the vehicle group and the two groups RTG (7.5 mg/kg, i.p., *p *< 0.01; and 2.5 mg/kg, i.p., *p *< 0.05, one-way ANOVA followed by Dunnett's test), and the morphine group (Mor) (5 mg/kg, i.p., *p *< 0.05, one-way ANOVA followed by Dunnett's test).

**Figure 7:**
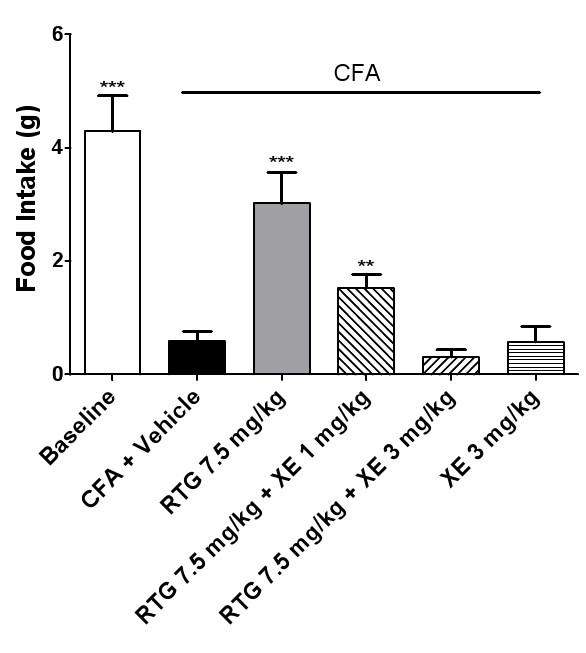
**Enhanced food intake by retigabine was reversed by the blocker XE991**. The food intake in the baseline group represents the value for normal rats without TMJ inflammation. The effect of retigabine or retigabine and XE991 was tested 24 hours after CFA injection. The food intake in the vehicle group indicates the value for rats with TMJ inflammation induced by CFA. Retigabine at a dose of 7.5 mg/kg, i.p. significantly increased the food intake, as compared with the vehicle group (*p *< 0.005, one-way ANOVA followed by Dunnett's test). Co-injection (i.p.) of XE991 (at 3 mg/kg or 1 mg/kg) with RTG reversed the food intake which had been increased by RTG (7.5 mg/kg, *p *< 0.05, one-way ANOVA followed by Dunnett's test), as compared with the vehicle group. XE991 at 3 mg/kg had no effect on food intake in rats with TMJ inflammation (p = 0.98), compared with vehicle group. RTG: Retigabine; XE: XE991.

In contrast, food intake was significantly decreased in rats with TMJ inflammation (Figure 6). Administration of retigabine in doses from 2.5 mg/kg to 7.5 mg/kg increased the food intake of TMD rats about 1.0 to 3.0-fold, as compared with the vehicle control group (Figure 6). Increased food intake associated with retigabine was reversed by XE991 at doses of 1 mg/kg or 3 mg/kg (Figure 7). These results were consistent with the results of measurement of elevated head withdrawal threshold, further confirming attenuation of mechanical allodynia in inflamed TMJs by retigabine through activation of the KCNQ/Kv7 channel function.

### Analgesic effect with central intracerebroventricular administration of retigabine

**Figure 8:**
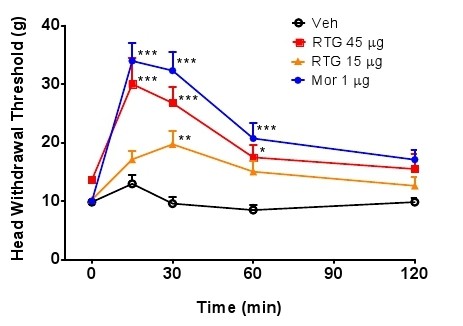
**Allodynia was attenuated by central administration of retigabine in rats with TMJ inflammation**. Intracerebroventricular injection of retigabine (45 μg or 15 μg) as well as morphine (1 μg) as positive control raised the head withdrawal threshold from 15 to 60 min, compared with the vehicle control (*p *< 0.05 two way ANOVA followed by Bonferroni post-test).

**Figure 9:**
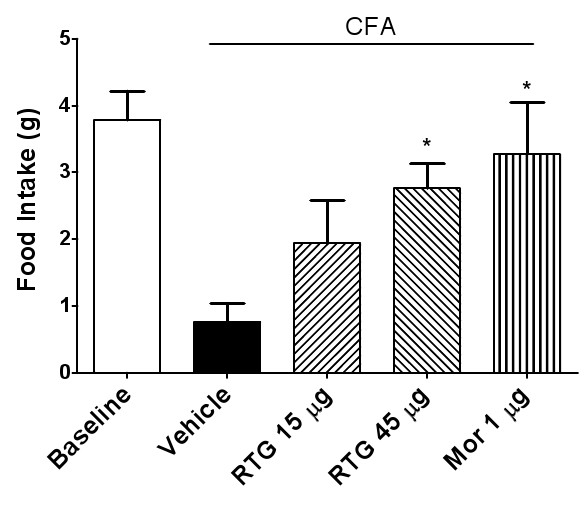
**Increased food intake secondary to central administration of retigabine**. Intracerebroventricular administration of retigabine at a dose of either 45 μg or 15 μg (*p *< 0.05), as well as the positive control morphine group 1 μg (*p *< 0.05, one-way ANOVA followed by Dunnett's test) raised the food intake in rats with TMJ inflammation.

In order to further confirm attenuation of allodynia with inflamed TMJs was due to reduction of central excitability by activation of KCNQ channels, we injected retigabine into the rats with TMJ inflammation intracerebroventricularly (i.c.v). Retigabine (45 or 15 μg) as well as the positive control morphine (1 μg) significantly elevated the mechanical head withdrawal threshold, as compared with the vehicle control (Figure 8). Similarly, food intake was also increased with icv injection of retigabine (15 μg) into rats with TMJ inflammation, as compared with the vehicle control (Figure 9). These results indicate that retigabine resulted in reduced allodynia in rats with TMJ inflammation, further confirming the analgesic effect of retigabine on TMJ inflammation through central suppression of neuronal hyperexcitability.

## Discussion

We were intrigued by the recent study in which two NSAIDs, the class of drugs which are the first line clinical treatment for TMD pain, were shown to suppress epileptic activities by activation of neuronal voltage-gated KCNQ/Kv7 channels [24]. This observation led us to hypothesize that the anticonvulsant retigabine that specifically opens KCNQ/Kv7 channels might be beneficial for TMD pain. In this study for the first time, we have shown that central activation of neuronal KCNQ/Kv7 potassium channels by retigabine can attenuate allodynia associated with temporomandibular joint inflammation in rats. Our findings have several implications: first, they serve as an in vivo validation that neuronal KCNQ/Kv7 potassium channels are a target for TMJ pain; second, the positive modulation of neuronal KCNQ/Kv7 potassium channels that suppress excessive excitability can be therapeutically beneficial to TMDs; third, the anticonvulsant retigabine which is effective in clinical trials for reduction in seizures may also prove to be useful in pharmacological intervention in TMDs; finally, central hyperexcitability is involved in the mechanism of inflammatory TMJ pain.

TMD pain is considered to be a disorder characterized by central hyperexcitability and sensitization, and this is further supported by the fact that central-acting pharmacological agents including the anticonvulsant gabapentin show clinical efficacy for analgesia and anti-hyperalgesia in treatment of TMDs [7,8]. Neuronal hyperexcitability is a common underlying mechanism of neurological disorders such as epilepsy and chronic pain including TMDs. These disorders are currently managed by drugs that are dampen neuronal hyperexcitability through voltage-gated sodium channel inhibition, modulation of voltage-gated calcium channels and their auxiliary subunits, and inhibitory GABAergic neurotransmission [6]. Although the cause of excessive neuronal activity varies, an increase in voltage-gated K^+ ^channel conductance can suppress the hyerexcitability, thus providing a therapeutic potential for chronic pain. The voltage-gated potassium channels play a common role in repolarizing membrane potential of neurons during action potential firing. Typical neuronal firing is characterized by a specific voltage threshold for action potential, and the opening of potassium channels below the threshold (or subthreshold) will lead to the inhibition of initiation and propagation of action potential.

Head withdrawal is a response to nociceptive stimuli applied to the facial skin, and head withdrawal threshold is regulated by excitability of TRG neurons innervating the TMJ [32]. It has been shown that voltage-gated transient potassium (Kv) current regulates the excitability of TRG neurons, and the Kv channel density is lower in inflamed TMJ, leading to increased excitability of TRG neurons [25,33]. In terms of action mechanism of retigabine, there is a lack of literature report as to whether KCNQ/M-current is expressed in TRG neurons, and our data can not rule out retigabine partially working on peripheral TRG neurons for activation of Kv current.

The existence of a low-threshold, depolarization activated potassium current was described in 1980 and is referred to as the "M-current" because it was inhibited by the cholinergic agonist muscacine [34,35]. The M-current is active in the voltage range for action potential initiation and is therefore of particular importance in regulating the dynamics of the neuronal firing of nociceptive neurons and excitability of C-type nerve fibers [14,15]. The M-current turns on slowly following membrane depolarization and does not inactivate with sustained depolarization. The molecular identity that underlies the M-current was discovered as a result of identification of mutations in human potassium channel subunits referred to as KCNQ2 (Kv7.2) and KCNQ3 (Kv7.3) from idiopathic generalized epilepsy patients [36,37]. It was demonstrated that the M-current channel is formed by KCNQ2 and KCNQ3 subunits as heteromultimers as well as homomultimers [17]. M-current can also possibly be generated by heteromultimers with other KCNQ subunits such as KCNQ5 [38]. The expression of KCNQ subunits has also been confirmed in the CNS and DRG sensory neurons [10,39,40], suggesting the role the channels play in regulating neuronal excitability.

There are no previously published studies in the literature evaluating the analgesic effect of retigabine on TMJ pain. Pharmacological treatment of TMDs remains a clinical challenge because of diverse etiologic factors that contribute to the severity of TMDs pain. Without a clear rationale based on mechanism for selection of drugs, a wide-spectrum of available drugs such as NSAIDs, antidepressants, benzodiazepines, muscle relaxants, corticosteroids, anticonvulsants and opioids etc, has been used to achieve desired therapeutic end points for TMDs. Accumulating clinical evidence shows that patients with TMDs have generalized central hypersensitivity, suggesting that anticonvulsant drugs may have therapeutic potential [1,5,8]. Retigabine is a promising new anticonvulsant that has been shown to have a broad-spectrum of activity in animal models of epileptic seizures with a recently described novel mechanism of action which involves specific activation of KCNQ2-5 (Kv7.2-7.5) channels [9,41-43]. There are preliminary indications that in the rat the half-life and concentrations of retigabine are higher in the brain than in the plasma [43]. Retigabine has also been shown to relieve hyperalgesia and allodynia in animal models of neuropathic pain [9,10]. In previous studies of rotarod test that evaluates balancing and coordination for CNS side effect [12], a dose of 10 mg/kg retigabine was given to rats intraperitoneally to test its side effects of central nervous system. The administration of retigabine caused a short period of impaired motor performance, although the motor performance of all rats returned to baseline level 15 min after administration of the drug [12]. In order to avoid performance impairment, we reduced the dose to 7.5 mg/kg, and no significant performance impairment was observed during the behavior test in our study. We also tested the effect of retigabine and XE991 on normal rats, and we did not observe any abnormal pain sensation or abnormality of food intake with these agents. This result indicates that that retigabine preferentially functions in hyperactive neurons, which is consistent with the observation that the effect of retigabine is profound in depolarized neurons and small in hyperpolarized axons [14,15]. Taken these findings together, the pharmacological effect of retigabine on TMJ inflammation was specific, and was not due to any toxic effect [43].

## Conclusions

This study shows the analgesic effect of retigabine operates through specific activation of neuronal KCNQ/Kv7 in TMDs in rats. Because of the central acting property of retigabine, our results indicate that the central hypersensitivity of TMD patients may be an important mechanism in this disease. Therefore, the reduction of central hyperexcitability by anticonvulsant agents may represent a potential therapeutic alternative for TMDs.

## Methods

### Animals

Adult male Sprague Dawley rats (190-220 g) were used in this study. The experimental protocols were approved by the Animal Use and Care Committee of Peking University and were consistent with the Ethical Guidelines of the International Association for the Study of Pain. The rats were housed under controlled temperature (22 ± 1°C), on a 12 hr light/dark cycle and had free access to food and water.

### Induction of temporomandibular joint (TMJ) inflammation by complete Freund's adjuvant (CFA)

Because TMD pain is significantly related to synovitis, internal derangement and osteoarthritis, indicating that joint inflammation could be a major reason for TMD pain, TMJ inflammation is used universally to mimic TMD pain [44-47]. After baseline measurements of head withdrawal threshold and food intake (see below) were completed, rats were briefly sedated with chloral hydrate (300 mg/kg) and then 50 μl of CFA suspension (1:1, CFA fluid/saline suspension) (Sigma, St. Louis, MO, USA) per side were injected into the bilateral TMJ with a 30-gauge needle. For the control rats, 50 μl of saline per side were injected into the bilateral TMJs [26,27]. TMJ inflammation was evaluated by physical examination and histopathology.

### Tissue preparations and staining of TMJ

The TMJ was removed and fixed in 4% paraformaldehyde in phosphate buffered solution (PBS) and demineralized in 15% EDTA. The specimens were dehydrated in graded alcohols and xylene, embedded in paraffin, and cut serially into 5-μm sagittal sections. The sections were stained with hematoxylin-eosin. After the staining, the sections were imaged using Olympus BX60 system microscope (Olympus Corp., Tokyo, Japan) containing ApogeeKX85 digital camera (Apogee Biotechnology Corporation, USA) and the images were acquired by Image Pro Plus software (Media Cybernetics, Inc., USA) without any subsequent image manipulation.

### Measurement of head withdrawal threshold

At least 1 week before the mechanical nociceptive threshold testing, the rats were housed in the testing room with 3-4 animals per cage. The head withdrawal threshold was measured as previously reported [27]. Briefly, 24 h after injection of CFA into the TMJ, the rats were habituated to lean against the experimenter's gloved hand (regular leather working glove) standing on their hind paws. During the test session, the rats were unrestrained but remained motionless. A filament conveying progressive, increasing forces from an electronic von Frey Anesthesiometer (IITC Life Science, CA, USA) was applied to the bilateral TMJ regions until the head was withdrawn and the applied force was recorded. The combined head withdrawal threshold measured from both sides of TMJ regions was calculated as mean ± standard error of mean (s.e.m.) based on at least five measurements per joint with 6-8 rats per group.

### Food intake

Food intake is negatively associated with TMJ inflammation/pain and can be used as an indicator of TMJ inflammation/pain [26,30,31]. We measured food intake as previously described with minor modifications, whereby rats were initially fasted over a period of time and then the amount of food subsequently eaten was measured over a limited interval. Briefly, after injection of CFA into the TMJ, each rat was kept in one cage supplied with water but without food for 12 h. The rat was then fed with food but without water and the amount of food eaten by the rat during 2 h was recorded as the food intake. After drug treatment, a substantial quantity of food was put into each cage, and the initial amount of food was the same in every cage. This food was carefully kept dry during the test session to avoid artefactual weight alteration, and the residual food was weighted. The difference between the initial and residual amount of food was recorded as the amount of food intake during 2 hours.

### Drugs administration

Before behavioral testing, the anticonvulsant retigabine or the KCNQ/Kv7 channel blocker XE991 (Tocris, UK) was dissolved in tween 80 (Sigma, St. Louis, MO, USA) and saline. The vehicle used in this study was a mixture of tween80 (Sigma, St. Louis, MO, USA) and saline (0.9% NaCl, autoclaved before use) in a ratio of 1:9 (v/v). Drugs were diluted to desired concentrations one day before the experiments and were stored at -20°C. All drug solutions were administered to rats intraperitoneally at a volume of 10 ml per kilogram body weight. Intraperitoneal (i.p.) injection of drug solutions and behavior testing were conducted on the basis of a double-blind and randomized design, in which one experimenter took the charge of drug injection and randomized rats dividing, whereas another experimenter who was blind to drug administration and grouping, conducted the measurements of head withdrawal threshold and food intake.

### Intracerebroventricular catheterization and injection

Surgery was carried out as previously described [48]. Briefly, the anesthetized rat was placed in a stereotaxic frame. A skin incision was made on the skull and the bregma was located. An i.c.v. guide cannula (21-gauge) was inserted stereotaxically (coordinates: 0.6 mm caudal to bregma, 1.6 mm lateral to the midline, 1.8 mm beneath the surface of the skull). Two screws were attached to the skull with dental cement (Harward Dental GmbH, Berlin, Germany), securing the catheter to the skull. After the experiment the position of the catheter was examined. The rat was sacrificed by intraperitoneal chloral hydrate. Immediately after death, dye (30 ul 1% methylene blue) was injected intracerebroventricularly. To confirm the accuracy of catheterization and injection, the coronal sections (30 μm) of the brain were sliced on a cryostat and the perforative lesion with blue dye were observed in the cavity and wall of cerebroventricle under the examination of microscopy. Retigabine and morphine were dissolved in Dimethyl sulfoxide (DMSO)(Sigma, St. Louis, MO, USA) in desired concentration 1 day before the experiment. The drugs were given in a volume of 2 ul and the pure DMSO as control was administered in vehicle group. The behavior experiments were conducted on the basis of a double-blind and randomized design as described in above section of drug administration.

### Data analysis

Statistical analysis was performed with GraphPad Prism (the 5th edition, GraphPad Software, USA) for Windows. All data were presented as mean ± s.e.m. Statistical significance between multiple groups was examined by one or two-way ANOVA with appropriate post hoc test (see figure legends for details). A value of *p *< 0.05 was considered to be statistically significant.

## Abbreviations

ANOVA: analysis of variance; CFA: complete Freund's adjuvant; CNS: central nervous system; COXs: cyclooxygenases; DMSO: dimethyl sulfoxide; DRG: dorsal root ganglion; GABA: gamma-aminobutyric acid; NSAIDs: nonsteroidal anti-inflammatory drugs; RTG: retigabine; S.E.M.: standard error of mean; TMD: temporomandibular disorders; TMJ: temporomandibular joint.

## Competing interests

The authors declare that they have no competing interests.

## Authors' contributions

WX carried out behavioral assays, animal surgery, imaging and histological experiments and drafted the manuscript. YPB, LT, and YWW participated in double blind behavioral experiments. YPB and YWW helped with animal surgery, histological and imaging experiments. KWW designed and finished the final draft of the manuscript. All authors read and approved the final manuscript.
